# Predictive value of pre-existing thyroid autoantibodies for PD-1 inhibitor-induced hypothyroidism: a retrospective cohort study

**DOI:** 10.3389/fendo.2026.1905074

**Published:** 2026-09-18

**Authors:** Peiying Huang, Huangdao Yu, Xuanling Zheng, Mingzhu Lin, Fangsen Xiao, Xuejun Li, Jinyang Zeng, Fangfang Yan

**Affiliations:** 1 Xiamen First Clinical Medical College of Fujian Medical University, Xiamen, China; 2 Department of Endocrinology and Diabetes, the First Affiliated Hospital of Xiamen University, School of Medicine, Xiamen University, Xiamen, China; 3 Xiamen Diabetes Institute, Xiamen, China; 4 Department of Gastrointestinal Oncology Surgery, the First Affiliated Hospital of Xiamen University, School of Medicine, Xiamen University, Xiamen, China

**Keywords:** hypothyroidism, immune-related adverse event, PD-1 inhibitor, risk prediction, thyroid autoantibody

## Abstract

**Introduction:**

Immune checkpoint inhibitors (ICIs), particularly programmed cell death-1 (PD-1) inhibitors, have revolutionized cancer treatment but frequently induce thyroid dysfunction as the most common endocrine immune-related adverse event (irAE). Baseline thyroid autoantibodies have been implicated as potential risk factors, yet the dose-response relationship between antibody titers and hypothyroidism risk, and the differential predictive value of thyroperoxidase antibody (TPOAb) versus thyroglobulin antibody (TGAb) titers, remain incompletely characterized.

**Methods:**

We conducted a retrospective analysis of 627 patients with various malignancies who received PD-1 inhibitor therapy between 2015 and 2025. Thyroid function and autoantibodies (TPOAb, TGAb) were monitored every 3 weeks. Antibody titers were analyzed both as continuous variables and as ordinal strata. Univariate and multivariate logistic regression analyses were performed; receiver operating characteristic (ROC) curve analysis was used to determine optimal titer cutoffs, and a predictive model was constructed and evaluated. Kaplan-Meier survival analysis assessed prognostic implications.

**Results:**

Hypothyroidism occurred in 247 patients (39.4%), with onset at a median of 12.0 weeks. In multivariate analysis, baseline TPOAb positivity (OR = 3.681, 95% CI: 2.237–6.056, P < 0.001) and TGAb positivity (OR = 4.635, 95% CI: 2.335–9.200, P < 0.001) were independently associated with hypothyroidism risk. When analyzed as continuous variables, each 100 IU/mL increase in TPOAb conferred an OR of 2.699 (P < 0.001), and each 100 IU/mL increase in TGAb conferred an OR of 1.516 (P = 0.007). Dose-response analysis revealed progressive escalation of hypothyroidism rates with increasing antibody titers: for TPOAb, 30.7% (normal), 34.9% (mild, 10–49 IU/mL), 52.2% (moderate, 50–99 IU/mL), 69.6% (high, 100–499 IU/mL), and 77.5% (very high, ≥500 IU/mL); for TGAb, 34.1% (normal), 89.3% (mild, 80–199 IU/mL), 64.3% (moderate, 200–499 IU/mL), and 72.5% (high, ≥500 IU/mL). ROC analysis identified optimal titer cutoffs of 14.5 IU/mL for TPOAb (AUC = 0.647) and 11.5 IU/mL for TGAb (AUC = 0.588); the combined predictive model achieved an AUC of 0.718.

**Discussion:**

Baseline thyroid autoantibody titers demonstrate a clear dose-response relationship with hypothyroidism risk following PD-1 inhibitor therapy. TGAb positivity, even at mildly elevated titers, confers exceptionally high risk. The optimal TGAb cutoff of 11.5 IU/mL, substantially below conventional thresholds, suggests that even low-level TGAb may indicate clinically relevant subclinical autoimmunity.

## Introduction

Immune checkpoint inhibitors (ICIs) targeting programmed cell death-1 (PD-1) and its ligand, programmed cell death ligand 1 (PD-L1), have transformed the therapeutic landscape of multiple malignancies, offering durable antitumor responses in patients with advanced cancer (1, 2). However, the enhanced immune activation underlying their efficacy predisposes patients to immune-related adverse events (irAEs) that can affect virtually any organ system (3, 4). Among endocrine irAEs, thyroid dysfunction is the most frequently observed manifestation, with reported incidence rates ranging from 10.1% (5) to 40% (6, 7) depending on the ICI class and treatment regimen. The clinical spectrum typically encompasses a biphasic course characterized by transient thyrotoxicosis followed by persistent hypothyroidism, although isolated hypothyroidism without preceding hyperthyroidism is also commonly encountered (8).

The pathophysiology of ICI-induced thyroid dysfunction is thought to involve T-cell-mediated destructive thyroiditis, wherein activated cytotoxic T lymphocytes infiltrate thyroid tissue, leading to follicular cell damage and subsequent release of preformed thyroid hormones (9, 10). Several risk factors for thyroid irAEs have been identified, including baseline thyroid autoantibody positivity, elevated baseline thyroid-stimulating hormone (TSH) levels, prior tyrosine kinase inhibitor use, high body mass index, and heterogeneous thyroid echotexture (11, 12). Baseline positivity for thyroperoxidase antibodies (TPOAb) and/or thyroglobulin antibodies (TGAb) has emerged as one of the most consistently reported predictors.

Despite the accumulating evidence, several critical questions remain unanswered. First, most prior studies dichotomized antibody status as positive or negative without evaluating the dose-response relationship between antibody titers and the risk of hypothyroidism. Emerging evidence suggests that antibody titers, and not merely positivity status, may refine risk stratification. A prospective study demonstrated a dose-dependent risk: patients with severely elevated TPOAb titers (>500 IU/mL) experienced a 93.75% incidence of thyroid irAEs, compared to 60.0% for moderately elevated titers, and 13.33% for normal titers (13). Second, whether TPOAb and TGAb exert different dose-dependent effects on the risk of hypothyroidism remains controversial. Studies have reported that TGAb positivity is associated with a higher risk of thyrotoxicosis, whereas both TPOAb and TGAb positivity predicted hypothyroidism (14). Third, optimal titer cutoffs for clinical risk stratification have not yet been established.

Thus, we conducted a large-scale retrospective cohort study of 627 patients with diverse malignancies who received PD-1 inhibitor monotherapy, with systematic thyroid function monitoring every 3 weeks. Our primary objectives were to (1) evaluate the dose-response relationship between baseline TPOAb/TGAb titers and hypothyroidism risk; (2) determine optimal antibody titer cutoffs for risk stratification using receiver operating characteristic (ROC) curve analysis; (3) compare the differential predictive value of TPOAb versus TGAb as continuous versus categorical variables; (4) develop and validate a clinically applicable risk prediction model incorporating continuous antibody titers.

## Materials and methods

### Study design and population

The studies involving humans were approved by the Ethics Committee of the First Affiliated Hospital of Xiamen University, School of Medicine, Xiamen University (approval number: XMYY-2022KYSB160). The requirement for written informed consent was waived by the Ethics Committee due to the retrospective nature of the study using de-identified data. The studies were conducted in accordance with the local legislation and institutional requirements.

Eligibility criteria included: (1) age ≥18 years; (2) histologically or cytologically confirmed malignancy; (3) treatment with PD-1 inhibitor monotherapy; (4) availability of baseline thyroid function tests and autoantibody measurements within 2 weeks prior to ICI initiation; and (5) thyroid function assessments were performed every 3 weeks coinciding with ICI administration cycles after treatment initiation. Patients with pre-existing overt hypothyroidism requiring levothyroxine replacement were excluded unless they had a documented euthyroid status at baseline. Patients receiving combination ICI regimens (e.g., ipilimumab plus nivolumab) or those who had previously received radiotherapy to the head, neck, or thoracic region were excluded to minimize confounding factors. As part of institutional standard of care since 2015, all patients receiving PD-1 inhibitor therapy at our center underwent a standardized thyroid monitoring protocol implemented by the Endocrinology Consultation Service. This protocol required baseline thyroid function tests (TSH, FT3, FT4) and thyroid autoantibodies (TPOAb, TGAb) within 2 weeks prior to ICI initiation, followed by repeat testing every 3 weeks to coincide with scheduled ICI administration cycles. Compliance with the monitoring protocol was documented in the electronic medical record system.

### Thyroid autoantibody assays

Thyroid autoantibodies (TPOAb and TGAb) were measured using two different automated immunoassay platforms during the study period:

Platform 1 (January 2015 - June 2020): Roche Cobas e602 analyzer using electrochemiluminescence immunoassay (ECLIA; Roche Diagnostics, Mannheim, Germany). TPOAb reference range: <10 IU/mL; functional sensitivity: 5.0 IU/mL. TGAb reference range: <80 IU/mL; functional sensitivity: 10.0 IU/mL. Inter-assay coefficient of variation (CV): <5.0% for TPOAb and <6.5% for TGAb. Intra-assay CV: <3.5% for both assays.

Platform 2 (July 2020 - December 2025): Abbott Architect i2000SR using chemiluminescent microparticle immunoassay (CMIA; Abbott Laboratories, Abbott Park, IL, USA). TPOAb reference range: <5.61 IU/mL; functional sensitivity: 1.0 IU/mL. TGAb reference range: <4.11 IU/mL; functional sensitivity: 1.0 IU/mL. Inter-assay CV: <7.0% for TPOAb and <8.0% for TGAb. Intra-assay CV: <4.5% for both assays.

To ensure cross-platform comparability, all antibody titers obtained from Platform 2 (Abbott) were standardized to the Platform 1 (Roche) reference scale using manufacturer-provided conversion protocols. The conversion was verified by parallel testing of 50 clinical samples on both platforms, yielding strong correlations (Pearson r > 0.95) for both TPOAb and TGAb. All reported titers are expressed in Roche-equivalent units (IU/mL), and positivity thresholds (TPOAb ≥10 IU/mL; TGAb ≥80 IU/mL) were applied uniformly across the study period using the standardized values.

### Data collection and variable definitions

Demographic, clinical, and laboratory data were extracted from the electronic medical record system. The baseline characteristics included age, sex, smoking history, tumor histology, tumor stage(American Joint Committee on Cancer, 8th edition), Eastern Cooperative Oncology Group performance status, and baseline laboratory parameters. Thyroid function tests included free triiodothyronine (FT3), free thyroxine (FT4), TSH, TPOAb, TGAb, and TSH receptor antibody (TRAb).

The primary outcome was the development of hypothyroidism following PD-1 inhibitor initiation, defined as TSH >4.2 mIU/L with FT4 below the lower limit of normal, or TSH >10 mIU/L, regardless of FT4, on at least two consecutive measurements. The time of onset was calculated from the first ICI dose to the first abnormal TSH measurement. Thyroid autoantibody positivity was defined as TPOAb ≥10 IU/mL and/or TGAb ≥80 IU/mL at baseline (13). For dose-response analyses, antibody titers were analyzed as both continuous variables (per 10 IU/mL, and per 100 IU/mL increments) and as ordinal strata: for TPOAb, normal (<10 IU/mL), mild elevation (10–49 IU/mL), moderate elevation (50–99 IU/mL), high elevation (100–499 IU/mL), and very high elevation (≥500 IU/mL); for TGAb, normal (<80 IU/mL), mild elevation (80–199 IU/mL), moderate elevation (200–499 IU/mL), and high elevation (≥500 IU/mL).

### Statistical analysis

Categorical variables are expressed as frequencies and percentages and compared using the chi-square test or Fisher’s exact test, as appropriate. Continuous variables were tested for normality using the Shapiro-Wilk test and expressed as mean ± standard deviation (SD) or median with interquartile range (IQR), compared using the independent t-test or Mann-Whitney U test. Univariate logistic regression was performed to identify potential predictors of hypothyroidism, with antibody titers analyzed as continuous variables (per 10 IU/mL and per 100 IU/mL increments), dichotomous variables (positive vs. negative), and ordinal categorical variables (titer strata). Variables with P<0.10 in univariate analysis were entered into a multivariate logistic regression model using backward stepwise selection (likelihood ratio method) to identify independent risk factors. The odds ratios (ORs) with 95% confidence intervals (CIs) were calculated.

Optimal antibody titer cutoffs for hypothyroidism prediction were determined using receiver operating characteristic (ROC) curve analysis with the Youden index (maximum sensitivity + specificity - 1). The area under the ROC curve (AUC) was calculated for individual antibody titers and combined predictive model. Model calibration was assessed using the Hosmer-Lemeshow goodness-of-fit test. Spearman’s rank correlation was used to evaluate the correlation between antibody titers and hypothyroidism. Kaplan-Meier survival curves were constructed for overall survival (OS), defined as the time from ICI initiation to death from any cause or the last follow-up. Survival distributions were compared using log-rank tests. All statistical analyses were performed using SPSS (version 26.0; IBM Corp., Armonk, NY, USA) and R version 4.3.1 (R Foundation, Vienna, Austria). Statistical significance was defined as a two-sided P-value of < 0.05. Spearman's rank correlation was also used to evaluate the correlation between baseline antibody titers and peak TSH levels among patients who developed hypothyroidism.

## Results

### Patient characteristics

A total of 627 patients were included in the final analysis. The baseline characteristics of the patients are summarized in **Table 1**. The median age was 65.0 years (IQR: 58.0-73.0), and 399 patients (63.6%) were male. The most common malignancies were lung cancer (n=214, 34.1%), hepatocellular carcinoma (n=73, 11.6%), gastric cancer (n=68, 10.8%), colorectal cancer (n=52, 8.3%), and nasopharyngeal carcinoma (n=49, 7.8%). Most patients (n=476, 75.9%) had Stage IV disease. The median number of ICI treatment cycles was five (IQR: 4-6). In the non-hypothyroidism group (n=380), 46 patients (12.1%) were TPOAb-positive only; 14 patients (3.6%) were TGAb-positive only; and 21 patients (5.5%) were positive for both antibodies. In the hypothyroidism group (n=247), 65 patients (22.7%) were TPOAb-positive only; 22 patients (8.9%) were TGAb-positive only; and 56 patients (22.7%) were positive for both antibodies. The median follow-up duration was 15.6 months (IQR: 7.0-29.8). Thyroid function assessments were performed at each ICI administration cycle, yielding a total of approximately 12,000 individual measurements during the study period. All 627 patients had complete thyroid monitoring data during their treatment period. The median baseline TPOAb titer was 5.0 IU/mL (IQR: 5.0-18.0) in the entire cohort, 5.0 IU/mL (IQR: 5.0-5.0) in patients without hypothyroidism, and 15.8 IU/mL (IQR: 5.0-508.0) in patients with hypothyroidism (P<0.001). The median baseline TGAb titer was 5.0 IU/mL (IQR: 5.0-20.0) overall, 5.0 IU/mL (IQR: 5.0-5.0) in the non-hypothyroidism group, and 20.0 IU/mL (IQR: 5.0-2800.0) in the hypothyroidism group (P<0.001).

**Table 1:** Baseline characteristics of patients receiving PD-1 inhibitor therapy.

Characteristic	No hypothyroidism (n=380)	Hypothyroidism (n=247)	P-value
Age, years	65.2 ± 11.4	64.5 ± 10.8	0.452
Male sex, n (%)	233 (61.3)	166 (67.2)	0.104
Smoking history, n (%)	62 (16.3)	48 (19.4)	0.351
Stage IV disease, n (%)	283 (74.5)	193 (78.1)	0.296
Treatment cycles, median (IQR)	5 (4–6)	5 (4–6)	0.782
Lymphocyte percentage, %	20.5 ± 12.3	21.8 ± 13.1	0.214
WBC, ×10^9^/L	8.2 ± 4.1	8.5 ± 4.3	0.387
ALT, U/L	28.5 ± 35.2	31.2 ± 38.6	0.356
AST, U/L	28.3 ± 32.1	33.5 ± 41.2	0.089
GGT, U/L	85.4 ± 142.3	92.1 ± 156.8	0.564
Albumin, g/L	38.5 ± 6.2	37.8 ± 6.8	0.198
TPOAb titer, median (IQR), IU/mL	5.0 (5.0–5.0)	15.8 (5.0–508.0)	<0.001
TGAb titer, median (IQR), IU/mL	5.0 (5.0–5.0)	20.0 (5.0–2800.0)	<0.001
Baseline TPOAb positive (≥10), n (%)	67 (17.6)	121 (49.0)	<0.001
Baseline TGAb positive (≥80), n (%)	35 (9.2)	78 (31.6)	<0.001
Both antibodies positive, n (%)	21 (5.5)	56 (22.7)	<0.001
Tumor site			0.421
Lung cancer	128 (33.7)	86 (34.8)	
Liver cancer	43 (11.3)	30 (12.1)	
Gastric cancer	42 (11.1)	26 (10.5)	
Colorectal cancer	33 (8.7)	19 (7.7)	
Nasopharyngeal cancer	31 (8.2)	18 (7.3)	
Other sites	103 (27.1)	68 (27.5)	

Data are presented as mean ± standard deviation, median (interquartile range), or n (%). P-values were calculated using independent t-test, Mann-Whitney U test, Chi-square test, or Fisher’s exact test as appropriate. Abbreviations: WBC, white blood cell; ALT, alanine aminotransferase; AST, aspartate aminotransferase; GGT, gamma-glutamyl transferase; TPOAb, thyroperoxidase antibody; TGAb, thyroglobulin antibody; IQR, interquartile range.

### Risk factor analysis: dichotomous antibody status

In univariate logistic regression analysis, baseline TPOAb positivity (OR = 4.852, 95% CI: 3.095-7.607, P<0.001), TGAb positivity (OR = 6.474, 95% CI: 3.360-12.469, P<0.001), both antibodies positive (OR = 4.852, 95% CI: 3.095-7.607, P<0.001), and any antibody positivity (OR = 5.040, 95% CI: 3.536-7.184, P<0.001) were significantly associated with increased hypothyroidism risk. Additionally, older age (OR = 0.968 per year, 95% CI: 0.953-0.983, P<0.001), female sex (OR = 1.695, 95% CI: 1.186-2.421, P = 0.004), and elevated Aspartate Aminotransferase (ALT) (OR = 1.003, 95% CI: 1.001-1.005, P = 0.003) demonstrated significant associations (**Table 2A**).

**Table 2:** Logistic regression analysis for hypothyroidism risk following PD-1 inhibitor therapy.

A. Dichotomous antibody status (positive vs. negative)				
Variable	Univariate OR (95% CI)	P-value	Multivariate OR (95% CI)	P-value
Baseline TPOAb positive (≥10 IU/mL)	4.852 (3.095–7.607)	<0.001	3.681 (2.237–6.056)	<0.001
Baseline TGAb positive (≥80 IU/mL)	6.474 (3.360–12.469)	<0.001	4.635 (2.335–9.200)	<0.001
Both antibodies positive	4.852 (3.095–7.607)	<0.001	—	—
Age (per year)	0.968 (0.953–0.983)	<0.001	0.968 (0.951–0.985)	<0.001
Male sex	0.590 (0.413–0.843)	0.004	0.608 (0.416–0.887)	0.010
AST (per U/L)	1.003 (1.001–1.005)	0.003	1.003 (1.000–1.005)	0.019
Lung cancer	1.050 (0.752–1.466)	0.774	1.287 (0.873–1.896)	0.190

B. Continuous antibody titers			
Variable	OR (95% CI)	P-value	Interpretation
TPOAb (per 1 IU/mL)	1.010 (1.007–1.013)	<0.001	—
TPOAb (per 10 IU/mL)	1.104 (1.076–1.133)	<0.001	Each 10 IU/mL increase
TPOAb (per 100 IU/mL)	2.699 (2.054–3.546)	<0.001	Each 100 IU/mL increase
TGAb (per 1 IU/mL)	1.004 (1.001–1.007)	0.006	—
TGAb (per 10 IU/mL)	1.042 (1.011–1.073)	0.006	Each 10 IU/mL increase
TGAb (per 100 IU/mL)	1.516 (1.117–2.059)	0.007	Each 100 IU/mL increase
Spearman ρ (TPOAb)	0.284	<0.001	Correlation with hypothyroidism
Spearman ρ (TGAb)	0.202	<0.001	Correlation with hypothyroidism
Variable	OR (95% CI)	P-value	Interpretation
TPOAb (per 10 IU/mL)	1.104 (1.076–1.133)	<0.001	Each 10 IU/mL increase
TPOAb (per 100 IU/mL)	2.699 (2.054–3.546)	<0.001	Each 100 IU/mL increase
TGAb (per 10 IU/mL)	1.042 (1.011–1.073)	0.006	Each 10 IU/mL increase
TGAb (per 100 IU/mL)	1.516 (1.117–2.059)	0.007	Each 100 IU/mL increase
Spearman ρ (TPOAb)	0.284	<0.001	Correlation with hypothyroidism
Spearman ρ (TGAb)	0.202	<0.001	Correlation with hypothyroidism

C. Titer stratification analysis (multivariate-adjusted OR vs. normal)				
Titer stratum	n (%)	Hypothyroidism rate	Adjusted OR (95% CI)	P-value
TPOAb				
Normal (<10 IU/mL)	267 (42.6)	30.7%	Reference	—
Mild (10–49 IU/mL)	232 (37.0)	34.9%	1.196 (0.824–1.737)	0.348
Moderate (50–99 IU/mL)	23 (3.7)	52.2%	2.434 (0.999–5.930)	0.050
High (100–499 IU/mL)	56 (8.9)	69.6%	5.135 (2.986–8.832)	<0.001
Very high (≥500 IU/mL)	40 (6.4)	77.5%	7.405 (3.443–15.928)	<0.001
TGAb				
Normal (<80 IU/mL)	534 (85.2)	34.1%	Reference	—
Mild (80–199 IU/mL)	28 (4.5)	89.3%	13.611 (3.868–47.910)	<0.001
Moderate (200–499 IU/mL)	14 (2.2)	64.3%	3.384 (0.995–11.510)	0.051
High (≥500 IU/mL)	40 (6.4)	72.5%	4.857 (2.075–11.368)	<0.001

D. Receiver operating characteristic analysis				
Model/Predictor	AUC (95% CI)	Optimal cutoff	Sensitivity	Specificity
TPOAb alone	0.647 (0.603–0.691)	14.5 IU/mL	50.2%	74.0%
TGAb alone	0.588 (0.542–0.634)	11.5 IU/mL	49.0%	67.8%
Combined model*	0.718 (0.677–0.760)	0.381	60.7%	68.2%

OR, odds ratio; CI, confidence interval; AUC, area under the curve; TPOAb, thyroperoxidase antibody; TGAb, thyroglobulin antibody. *Combined model includes TPOAb titer, TGAb titer, age, sex, and AST. Hosmer-Lemeshow test: χ²=6.847, P=0.553. Multivariate model AIC=628.34, BIC=665.71. The model equation was: P = 1/(1 + e^(−z)), where z = 1.6535 − 0.0337 × Age − 0.5061 × Sex (male=1) + 0.0026 × AST + 0.0278 × (TPOAb/10) + 0.0020 × (TGAb/10). The model achieved an AUC of 0.718 (95% CI:0.677–0.760), with an optimal probability cutoff of 0.381.

In the multivariate logistic regression analysis, baseline TGAb positivity emerged as the strongest independent predictor (OR = 4.635, 95% CI: 2.335-9.200, P<0.001), followed by TPOAb positivity (OR = 3.681, 95% CI: 2.237-6.056, P<0.001). Older age (OR = 0.968, 95% CI: 0.951-0.985, P<0.001) and male sex (OR = 0.608, 95% CI: 0.416-0.887, P = 0.010) were independent protective factors, whereas elevated AST levels (OR = 1.003; 95% CI: 1.000-1.005, P = 0.019) remained significant risk factors (**Table 2A**).

### Dose-response analysis: continuous antibody titers

When analyzed as continuous variables, both TPOAb and TGAb titers demonstrated significant dose-response relationships with the risk of hypothyroidism. To facilitate clinical interpretation, we calculated the ORs per standardized increment; each 10 IU/mL increase in TPOAb was associated with an OR of 1.104 (95% CI: 1.076-1.133, P<0.001), and each 100 IU/mL increase conferred an OR of 2.699 (95% CI: 2.054-3.546, P<0.001). For TGAb, each 10 IU/mL increase was associated with an OR of 1.042 (95% CI: 1.011-1.073, P = 0.006), and each 100 IU/mL increase conferred an OR of 1.516 (95% CI: 1.117-2.059, P = 0.007). Spearman’s rank correlation analysis confirmed significant positive correlations between antibody titers and hypothyroidism: TPOAb ρ=0.284 (P<0.001), TGAb ρ=0.202 (P<0.001) (**Table 2B**).

### Titer stratification and risk escalation

To further characterize the dose-response relationship, we stratified patients by baseline antibody titer levels. There were 267 patients (42.6%) with normal TPOAb titers and 534 patients (85.2%) with normal TGAb titers. For TPOAb, hypothyroidism rates demonstrated progressive escalation across strata: 30.7% in the normal titer group (<10 IU/mL), 34.9% in the mild elevation group (10–49 IU/mL), 52.2% in the moderate elevation group (50–99 IU/mL), 69.6% in the high elevation group (100–499 IU/mL), and 77.5% in the very high elevation group (≥500 IU/mL). Compared to the normal titer group, multivariate-adjusted ORs were 1.196 (95% CI: 0.824-1.737, P = 0.348) for mild elevation, 2.434 (95% CI: 0.999-5.930, P = 0.050) for moderate elevation, 5.135 (95% CI: 2.986-8.832, P<0.001) for high elevation, and 7.405 (95% CI: 3.443-15.928, P<0.001) for very high elevation (**Table 2C**).

For TGAb, the dose-response pattern was even more pronounced. Hypothyroidism rates were 34.1% in the normal titer group (<80 IU/mL), 89.3% in the mild elevation group (80–199 IU/mL), 64.3% in the moderate elevation group (200–499 IU/mL), and 72.5% in the high elevation group (≥500 IU/mL). Compared with the normal titer group, the multivariate-adjusted ORs were 13.611 (95% CI: 3.868-47.910, P<0.001) for mild elevation, 3.384 (95% CI: 0.995-11.510, P = 0.051) for moderate elevation, and 4.857 (95% CI: 2.075-11.368, P<0.001) for high elevation (**Table 2C**). Notably, even mildly elevated TGAb titers (80–199 IU/mL) confer an exceptionally high risk, with nearly 90% of patients in this stratum developing hypothyroidism.

### Correlation between baseline antibody titers and hypothyroidism severity

Among patients who developed hypothyroidism, Spearman correlation analysis revealed a modest but statistically significant positive correlation between baseline antibody titers and peak TSH levels during follow-up (TPOAb: ρ=0.248, P<0.001; TGAb: ρ=0.240, P<0.001), indicating that higher baseline antibody titers were associated with greater biochemical severity of hypothyroidism. (**Figure 1**).

**Figure 1:**
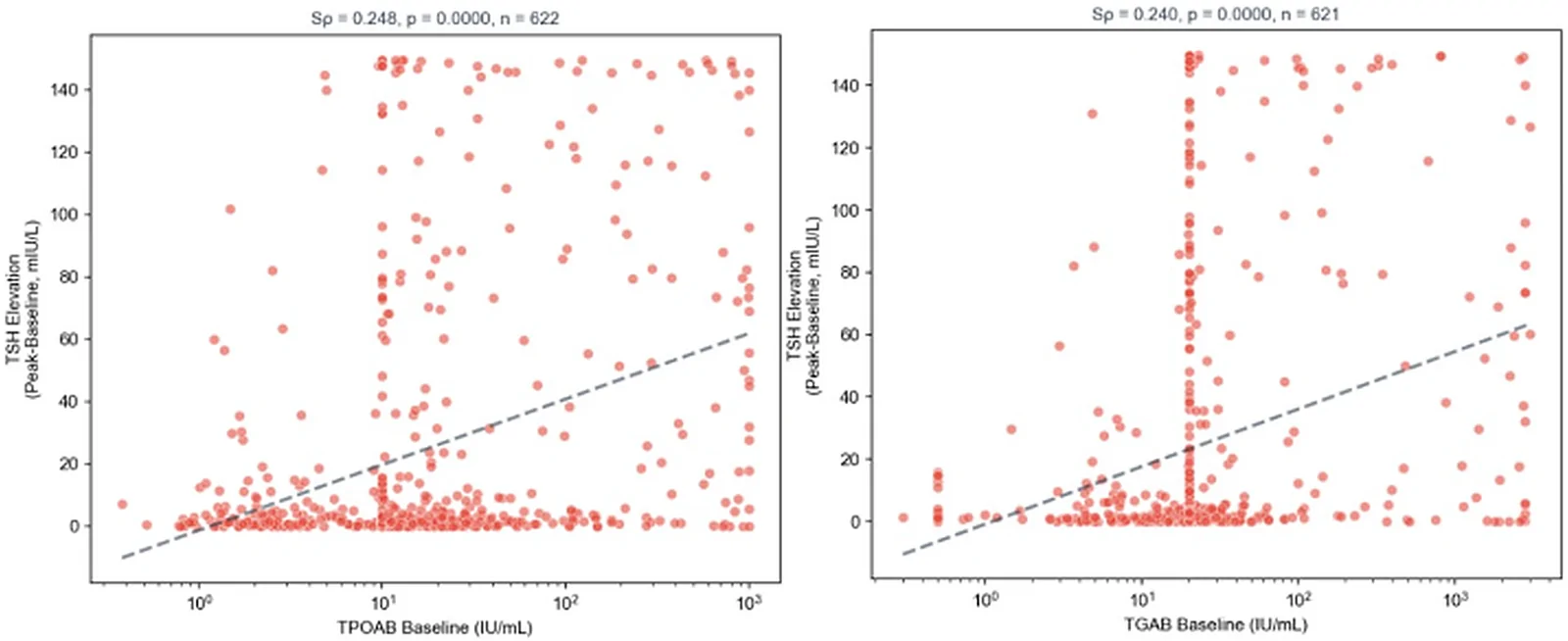
Correlation between baseline antibody titers and hypothyroidism severity.

### Optimal titer cutoff determination

ROC curve analysis was performed to identify the optimal antibody titer cutoffs for hypothyroidism prediction. For TPOAb, the AUC was 0.647 (95% CI: 0.603-0.691), with an optimal cutoff of 14.5 IU/mL (Youden index), yielding a sensitivity of 50.2% and specificity of 74.0%. For TGAb, the AUC was 0.588 (95% CI: 0.542-0.634), with an optimal cutoff of 11.5 IU/mL, yielding a sensitivity of 49.0% and specificity of 67.8%. When both antibody titers were combined with age, sex, and AST in a multivariate predictive model, the AUC improved to 0.718 (95% CI: 0.677–0.760) with an optimal probability cutoff of 0.381, achieving a sensitivity of 60.7%, a specificity of 68.2%, a positive predictive value of 54.8%, and a negative predictive value of 73.2% (**Figure 2**, **Table 2D**). The Hosmer-Lemeshow test indicated adequate calibration (χ²=6.847, P = 0.553).

**Figure 2:**
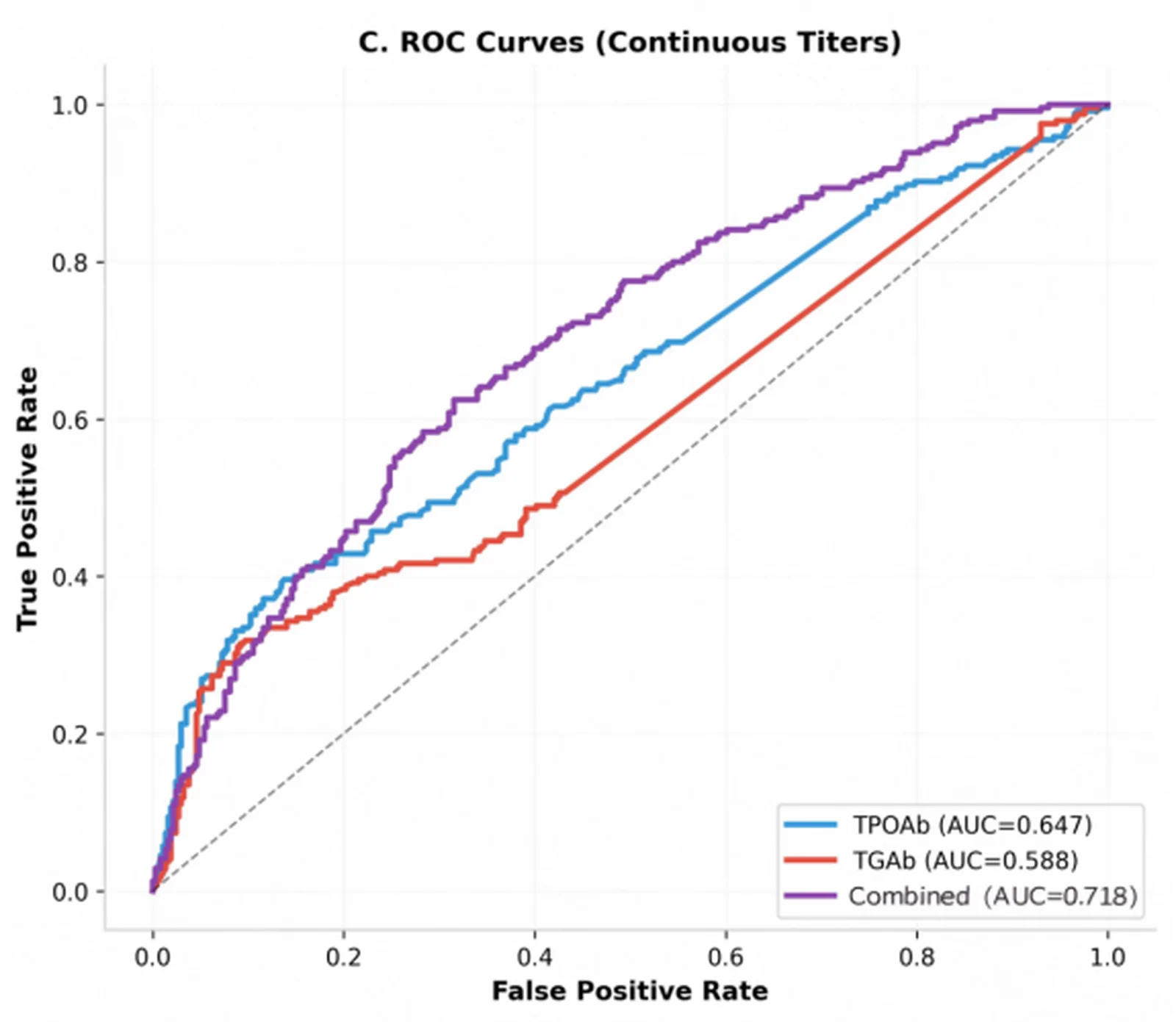
Receiver Operating Characteristic (ROC) curves for hypothyroidism prediction. ROC curves for TPOAb alone (blue, AUC=0.647), TGAb alone (red, AUC=0.588), and the combined predictive model (purple, AUC=0.718) incorporating continuous antibody titers, age, sex, and AST. The diagonal reference line (gray dashed) represents random chance. The optimal probability cutoff for the combined model was 0.381 (Youden index), with sensitivity=60.7% and specificity=68.2%.

### Survival analysis

Kaplan-Meier analysis revealed no significant difference in overall survival between patients who developed hypothyroidism and those who did not (log-rank P = 0.247) (**Figure 3A**). Similarly, the baseline thyroid antibody status did not significantly impact overall survival (log-rank P = 0.892) (**Figure 3B**). The median overall survival was not reached in either group during the observation period.

**Figure 3:**
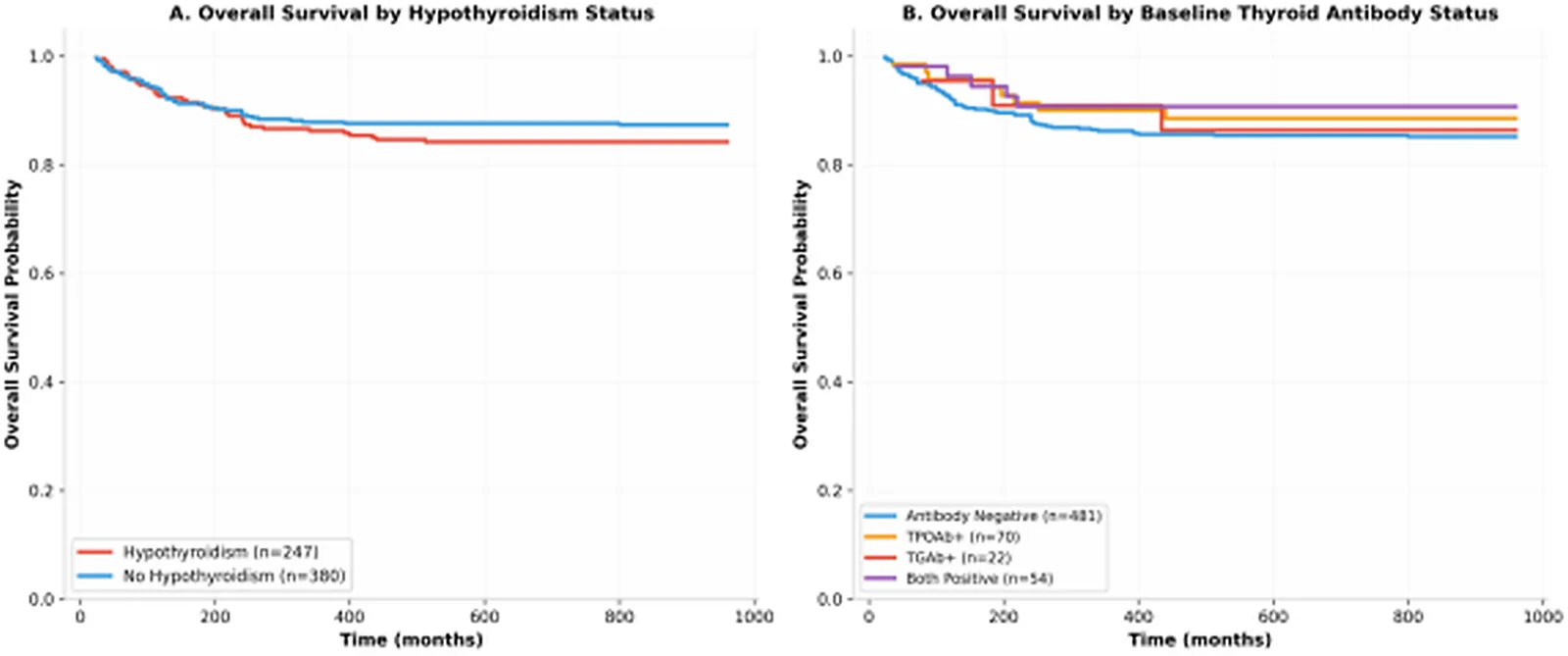
Kaplan-Meier survival curves for overall survival. **(A)** Overall survival stratified by hypothyroidism status (log-rank P=0.247). **(B)** Overall survival stratified by baseline thyroid autoantibody status (log-rank P=0.892).

## Discussion

This large retrospective cohort study of 627 patients with diverse malignancies undergoing PD-1 inhibitor therapy provides several important and novel insights into the dose-response relationship between baseline thyroid autoantibody titers and ICI-induced hypothyroidism. Our findings confirm that baseline thyroid autoantibody positivity is the most potent independent predictor of subsequent hypothyroidism, with TGAb positivity demonstrating a marginally stronger association than TPOAb positivity. Importantly, we demonstrated for the first time in a large cohort that antibody titers exhibit a clear dose-response relationship with hypothyroidism risk, with progressive escalation of risk across titer strata. Furthermore, we identified the optimal titer cutoffs for clinical risk stratification and developed a predictive model with moderate discriminative performance.

### Dose-response relationship between antibody titers and hypothyroidism risk

The most significant novel finding of our study is the demonstration of a robust dose-response relationship between baseline thyroid autoantibody titers and hypothyroidism risk. When analyzed as continuous variables, each 100 IU/mL increase in TPOAb conferred a 2.7-fold increased risk, whereas each 100 IU/mL increase in TGAb conferred a 1.5-fold increased risk. This finding extends beyond the simple positive/negative dichotomy employed in most previous studies and provides a more nuanced understanding of risk stratification. The dose-response pattern was particularly evident in our stratified analysis; for TPOAb, hypothyroidism rates escalated progressively from 30.7% (normal) to 34.9% (mild), 52.2% (moderate), 69.6% (high), and 77.5% (very high), with adjusted ORs increasing from 1.2 to 7.4 across strata. For TGAb, the pattern was even more striking, with mild elevation (80–199 IU/mL) conferring an exceptionally high risk of 89.3% and an adjusted OR of 13.6. Our cohort provided more granular risk estimates across multiple titer strata. The biological plausibility of this dose-response relationship is supported by the concept that higher baseline antibody titers reflect more extensive pre-existing autoimmune sensitization of the thyroid gland, which may be unleashed or amplified by PD-1 blockade-mediated T-cell reactivation.

### Differential predictive value of TPOAb versus TGAb

Our study revealed important differences between TPOAb and TGAb in their predictive value for hypothyroidism. While both antibodies were significant independent predictors, TGAb positivity conferred a higher univariate OR (6.47 vs. 4.85) and multivariate OR (4.64 vs. 3.68) than TPOAb positivity. This finding is consistent with a meta-analysis by Zhang et al., who identified a non-significant trend suggesting that TGAb positivity might confer a higher risk than TPOAb positivity (15). The differential predictive value may reflect distinct pathophysiological mechanisms: TPOAb targets thyroperoxidase, a key enzyme in thyroid hormone synthesis and the hallmark antibody in Hashimoto’s thyroiditis, whereas TGAb targets thyroglobulin, a precursor protein for thyroid hormone synthesis. The PD-1 blockade may preferentially activate follicular helper T cells, promoting B cell maturation and antibody class switching, potentially explaining the disproportionate effect of TGAb positivity. Additionally, the exceptionally high risk associated with mildly elevated TGAb titers (80–199 IU/mL) suggests that even a modest TGAb elevation may serve as a sensitive early indicator of subclinical autoimmune thyroid disease, which manifests clinically upon immune checkpoint blockade. The differential predictive value of TGAb versus TPOAb may reflect distinct immunological mechanisms. TGAb production requires cognate help from CD4+ T-follicular helper (Tfh) cells to thyroglobulin-specific B cells within germinal centers. PD-1 is highly expressed on Tfh cells and serves as a critical negative regulator of the germinal center response (16). PD-1 blockade may disproportionately disinhibit Tfh cells, leading to enhanced TGAb production and/or expansion of pre-existing TGAb-secreting B cell clones. In contrast, TPOAb production may depend less critically on Tfh-mediated help, potentially rendering it less sensitive to PD-1 pathway perturbation. Critically, circulating autoantibodies (TPOAb and TGAb) are principally biomarkers of pre-existing thyroid autoimmunity rather than direct mediators of follicular cell destruction. The actual tissue damage in ICI-induced thyroiditis is mediated primarily by cytotoxic CD8+ T cells that infiltrate the thyroid gland. Single-cell RNA sequencing studies have identified clonally expanded CXCR6+ IFN-γ+ cytotoxic CD8+ T cells within the thyroid of affected patients, a signature distinct from classical autoimmune thyroiditis (17). Therefore, baseline autoantibody positivity likely reflects an underlying state of T-cell autoreactivity primed for activation upon PD-1 blockade.

### Optimal titer cutoffs and clinical utility

Our ROC analysis identified optimal titer cutoffs of 14.5 IU/mL for TPOAb and 11.5 IU/mL for TGAb. Notably, both cutoffs were our findings have several significant clinical implications. Current guidelines recommend baseline thyroid function testing before ICI treatment initiation; however, antibody testing is not universally performed (19). Our data support the incorporation of baseline TPOAb and TGAb quantification into standard pretreatment workups, with titer-based risk stratification guiding monitoring intensity. Patients with TGAb ≥80 IU/mL or TPOAb ≥100 IU/mL may warrant particularly vigilant monitoring given their >70% risk of hypothyroidism. This finding suggests that even low-level TGAb positivity, below standard clinical cutoffs, may indicate subclinical thyroid autoimmunity that becomes clinically relevant under the immune-activating conditions of PD-1 blockade. PD-1 inhibitors disrupt peripheral tolerance mechanisms and may amplify pre-existing autoimmune responses, potentially conferring pathogenic significance to low-titer autoantibodies that would otherwise remain clinically silent. Clinically, this suggests that using the conventional ≥80 IU/mL threshold alone may miss a substantial number of at-risk patients. However, the modest AUC (0.588) and low specificity (67.8%) of the TGAb cutoff indicate that it should not be used in isolation. However, the modest AUC (0.588) and low specificity (67.8%) of the TGAb cutoff indicate that it should not be used in isolation. However, the modest AUC values for individual antibodies (0.647 for TPOAb and 0.588 for TGAb) indicate limited standalone predictive utility. The combined model incorporating both titers with demographic and clinical variables achieved a higher AUC of 0.718, representing moderate discriminative ability. Although inferior to a recently published nomogram incorporating TSH, antibody titers, and platelet counts (AUC = 0.863) (18), our model offers practical advantages by relying on routinely available parameters. The model’s negative predictive value of 73.2% suggests its utility for ruling out a high-risk status, potentially allowing for less intensive monitoring in low-risk patients.

### Demographic and clinical risk factors

Our multivariate analysis identified several independent demographic and clinical predictors. Older age was paradoxically associated with a reduced hypothyroidism risk (OR = 0.968 per year), which may reflect age-related immune senescence and attenuated T-cell reactivity following PD-1 blockade. This observation is consistent with the concept that robust immune reconstitution is a prerequisite for the development of irAEs (20). Male sex was independently protective (OR = 0.608), consistent with the well-established female predominance in autoimmune thyroid disease, and potentially reflects sex-specific differences in immune regulation, including X-chromosome-encoded immune genes and estrogen-mediated immune modulation (21). Elevated baseline AST levels emerged as a significant risk factor, possibly reflecting underlying hepatic immune activation or subclinical liver injury, which may parallel thyroid-directed autoimmunity.

### AST as an independent predictor

AST emerged as an independent predictor of hypothyroidism risk in our multivariate model (OR=1.003 per U/L, P=0.019). AST was included in our comprehensive panel of baseline laboratory variables because hepatic irAEs—particularly immune-mediated hepatitis manifesting as aminotransferase elevation—represent one of the most common and clinically significant toxicities associated with PD-1 inhibitor therapy. The association between elevated AST and subsequent hypothyroidism may reflect shared immunological pathways: both hepatic and thyroid irAEs involve T-cell-mediated tissue injury, and PD-1/PD-L1 signaling plays a critical role in maintaining peripheral tolerance in both organs. Subclinical hepatic immune activation, manifested as mild AST elevation, may parallel or precede thyroid-directed autoimmunity, with AST serving as an accessible surrogate marker of systemic immune activation. We note that the effect size for AST is modest, and this finding requires validation in independent cohorts. Future prospective studies incorporating comprehensive hepatic-immune biomarker panels, including cytokine profiling and liver immunophenotyping, are warranted to elucidate the mechanistic links between hepatic enzyme elevation and endocrine irAEs.

### Survival implications

The absence of survival differences between patients with and without hypothyroidism is noteworthy. Although some studies have suggested that thyroid irAEs may be biomarkers of enhanced antitumor immunity and improved prognosis in cancer (22, 23), our findings indicate that hypothyroidism itself does not confer a prognostic advantage across diverse malignancies. This may reflect the heterogeneity of our cohort, predominantly late-stage disease, or the fact that thyroid irAEs represent a nonspecific byproduct of immune activation rather than a surrogate for tumor-directed immunity. Importantly, the lack of a survival effect underscores the fact that hypothyroidism is primarily a manageable toxicity rather than a prognostic biomarker in our population. Single-cell RNA sequencing studies have elucidated the local immune landscape of ICI-induced thyroiditis. For instance, clonally expanded CXCR6^+^ IFN-γ^+^ cytotoxic CD8^+^ T cells have been identified within the thyroid of affected patients, a signature distinct from classical autoimmune thyroiditis (17). While our survival analysis showed no association with baseline TPOAb/TGAb positivity, single-cell approaches may clarify whether specific local T-cell clonal expansions, rather than circulating autoantibodies, determine clinical outcomes. Therefore, integrating single-cell transcriptomics with longitudinal antibody monitoring may reveal the immunopathological heterogeneity underlying thyroid irAEs. While our survival analysis showed no association with baseline TPOAb/TGAb positivity, single-cell approaches such as those by Lechner et al. (15) may clarify whether specific local T-cell clonal expansions, rather than circulating autoantibodies, determine clinical outcomes. Therefore, integrating single-cell transcriptomics with longitudinal antibody monitoring may reveal the immunopathological heterogeneity underlying thyroid irAEs.

### Strengths and limitations

The principal strengths of this study include the large sample size, systematic 3-week thyroid monitoring protocol, inclusion of diverse malignancies to enhance generalizability, and comprehensive evaluation of antibody titers as both continuous and stratified variables. To the best of our knowledge, this is the largest study to date to evaluate the dose-response relationship between thyroid antibody titers and ICI-induced hypothyroidism.

However, several limitations of this study must be acknowledged. First, the retrospective design introduced inherent selection and information biases. Second, its single-center nature may limit the external validity, particularly given the geographic variations in the prevalence of thyroid autoimmunity. Third, the antibody assays were performed using different platforms over the study period, potentially introducing measurement variability; however, all assays used consistent reference ranges. Fourth, T-cell data were available for only 8% of the patients, substantially limiting the statistical power of the immunological correlates. Fifth, we could not distinguish between primary hypothyroidism and the hypothyroid phase of biphasic thyroiditis in all cases. Sixth, the lack of an external validation cohort precludes a definitive assessment of the model generalizability. Finally, our optimal cutoff values require prospective validation prior to clinical implementation. The prevalence of thyroid autoantibody positivity varies substantially across populations, with reported rates ranging from approximately 10% in iodine-sufficient coastal regions to over 20% in iodine-deficient inland areas of China (24). Dietary iodine intake, a well-established modulator of thyroid autoimmunity, varies geographically in China and may influence baseline antibody prevalence (24). These geographic differences may limit the generalizability of our titer cutoffs and risk estimates to populations with substantially different background rates of thyroid autoimmunity (25, 26). Although antibody assays were performed using two different platforms over the study period, cross-platform standardization was performed; however, residual measurement variability cannot be completely excluded.

## Conclusion

In this large retrospective cohort study, baseline thyroid autoantibody titers demonstrated a clear and robust dose-response relationship with hypothyroidism risk following PD-1 inhibitor therapy. TGAb positivity, even at mildly elevated titers, confers exceptionally high risk.

## Data Availability

The original contributions presented in the study are included in the article/supplementary material. Further inquiries can be directed to the corresponding author/s.
